# Comparing Methods for Targeted Axillary Dissection in Breast Cancer Patients: A Nationwide, Retrospective Study

**DOI:** 10.1245/s10434-023-13792-x

**Published:** 2023-07-03

**Authors:** Frederikke Munck, Pernille Jepsen, Pernille Zeuthen, Lena Carstensen, Katrine Hauerslev, Christian K. Paaskesen, Inge S. Andersen, Ute Høyer, Charlotte Lanng, Maria K. Gerlach, Ilse Vejborg, Niels T. Kroman, Tove H. F. Tvedskov

**Affiliations:** 1 Department of Breast Surgery, Herlev-Gentofte Hospital, Hellerup, Denmark; 2 Department of Breast Surgery, Zealand University Hospital Roskilde, Roskilde, Denmark; 3 Department of Surgery and Plastic Surgery, Lillebaelt Hospital, Vejle, Denmark; 4 Department of Surgery Esbjerg, Hospital of South West Jutland, Esbjerg, Denmark; 5 Department of Plastic and Breast Surgery, Aarhus University Hospital, Aarhus, Denmark; 6 Research Unit for Plastic Surgery, Odense University Hospital, Odense, Denmark; 7 Department of Breast Surgery, Viborg Regional Hospital, Viborg, Denmark; 8 Department of Plastic and Breast Surgery, Aalborg University Hospital, Aalborg, Denmark; 9 Department of Pathology, Herlev-Gentofte Hospital, Hellerup, Denmark; 10 Department of Breast Examinations and Capital Mammography Screening, Herlev-Gentofte Hospital, Hellerup, Denmark

## Abstract

**Background:**

Several techniques exist for performing targeted axillary dissection (TAD) after neoadjuvant chemotherapy with the removal of the sentinel node and a marked metastatic lymph node (LN). Two-step methods include coil-marking of the metastatic LN at diagnosis and re-marking with an intraoperatively identifiable marker before surgery. Because nondetection of the marked lymph node (MLN) warrants axillary clearance and many patients achieve axillary pathological complete response (ax-pCR), the success of TAD is crucial. We compare various two-step TAD methods in a Danish national cohort.

**Methods:**

We included patients who received two-step TAD between January 1, 2016 and August 31, 2021. Patients were identified from the Danish Breast Cancer Group database and cross-checked with locally accessible lists. Data were extracted from the patient’s medical files.

**Results:**

We included 543 patients. In 79.4%, preoperative, ultrasound-guided re-marking was possible. Nonidentification of the coil-marked LN was more likely in patients with ax-pCR. The second markers used were hook-wire, iodine seeds, or ink marking on the axillary skin. Of patients with successful secondary marking, the MLN identification rate (IR) was 91%, and the sentinel node (SN) IR was 95%. Marking with iodine seeds was significantly more successful than ink marking with an odds ratio of 5.34 (95% confidence interval 1.62-17.60). The success rate of the complete TAD with the removal of MLN and SN was 82.3%.

**Conclusions:**

With two-step TAD, nonidentification of the coiled LN before surgery is frequent, especially in patients with ax-pCR. Despite successful remarking, the IR of the MLN at surgery is inferior to one-step TAD.

Axillary staging in a neoadjuvant setting has been a field of rapid development in the past few years, and no clear consensus has been established.^1,2^ The development was sparked by unacceptably high false-negative rates (FNR) in the range of 8.0-14.2% when the axilla was staged by sentinel lymph node biopsy (SLNB) after neoadjuvant chemotherapy (NACT).^3–7^ Today, some breast surgery centers still recommend SLNB as a staging procedure provided they use dual tracer and removal of three or more sentinel nodes (SNs), as this lowers the FNR.^8^ However, only 32.0-56.4% of patients staged by SLNB have three or more SNs identified.^3,6,9,10^ The development of a less-invasive staging technique for lymph node-positive patients receiving NACT seems imperative, considering that 31.4-63.0% of patients have axillary complete pathologic response (ax-pCR).^3,5,11–13^ These patients do not benefit from axillary lymph node dissection (ALND) but are nevertheless imposed to the possible morbidity associated with the procedure.

In a developmental effort, marking the metastasis-bearing lymph node before NACT and selective removal of the marked lymph node (MLN) upon surgery has been investigated. Straver et al. and Caudle et al. demonstrated the feasibility of this approach in 15 and 12 patients, respectively.^14,15^ They both reached an identification rate (IR) of 100%. Following this, the MARI procedure (excision of MLN guided by long-term radioactive seeds of iodine) was expanded to include 100 patients and demonstrated an IR of 97%. Despite a very high IR, the negative predictive value (NPV) was only 83%.^16^

This resulted in the selective removal of a marked node in combination with SLNB, and the combined staging technique is now known as Targeted Axillary Dissection (TAD). In several studies, TAD has demonstrated an FNR of 2.0-8.7%.^11,13,17^ The procedure is now widely used, but reports of technical challenges related to the identification of the marked node emerged, and the procedure is highly heterogeneous in terms of marking methods. Currently, reported IR ranges from 78-100%.^16,18–21^

Today, centers base the choice of marking method on preference and local legislation governing implants for use in patients. Few marking techniques exist that use a marker placed before NACT that is directly identifiable at surgery (one-step TAD). Alternatively, the more widespread two-step TAD is used with re-marking a coil-marked lymph node shortly before surgery, using a second marker identifiable in a surgical setting. Which marking method is superior in terms of IR of the MLN is yet unclear. So far, little evidence comparing different marking methods exists. Nonidentification of the MLN warrants ALND, and a procedure that ensures the highest possible IR is essential. This study compares two-step marking methods currently used in Danish Breast Surgery Departments.

## Materials and Methods

### Patient Identification

The study design is a nationwide, retrospective, cohort study. Patients were identified from the Danish Breast Cancer Group (DBCG) database. DBCG collects clinical and histopathological data and information regarding treatment plans and follow-up on all Danish women treated for breast cancer since 1977. The DBCG database identified patients receiving NACT between January 1, 2016 and August 31, 2021. Patients were stratified by surgical department.

All Danish breast surgery departments were subsequently contacted for information on the marking method used in these patients. Eight of nine departments agreed to participate. The one department declining participation cited not treating eligible patients as a reason.

### Inclusion and Exclusion Criteria

Patients were eligible for inclusion if they had a positive axillary lymph node at the diagnostic workup. A marking procedure of the positive lymph node was mandatory as well as an attempt at TAD at curative intended surgery (two-step marking procedure). Exclusion criteria comprised a history of ipsilateral axillary surgery for breast cancer, less than four cycles of NACT, a history of ipsilateral breast cancer, and patients staged by one-step marking procedures.

### Radiology

In all departments, breast radiologists examined patients at the time of diagnosis and performed breast and axillary ultrasonography (US), biopsy, and mammogram. Visually or palpably suspicious lymph nodes were biopsied either by fine-needle aspiration cytology (FNAC) or core-needle biopsy (CNB). Lymph nodes were considered positive if histo-/cytopathological evaluation of the biopsy returned “malignant cells,” “cells suspicious of malignancy,” or carcinoma originating from the breast. Marking of the positive lymph node was performed before or during the first few series of NACT. An axillary US with the placement of an intraoperatively identifiable marker was performed in the last weeks before surgery or immediately before surgery in the case of hook-wires.

### Surgery

TAD surgery consisted of excision of the MLN and SLNB. Using both a blue dye tracer and a radioactive colloid is recommended for SLNB in Danish guidelines.

### Data Collection

Patients identified in the DBCG database were cross-checked with each patient’s medical file and pathology and radiology reports. Data were registered in a REDCap database (Research Electronic Data Capture, vers. 10.6.18, 2022 Vanderbilt University, Nashville, TN 37240).^22,23^ Two departments provided local lists of patients receiving NACT (Department of Breast Surgery, Herlev Hospital) or lymph node marking (Department of Radiology, Rigshospitalet), which were cross-checked with the DBCG registration to decrease the risk of missing patients.

The following variables were registered: patient age, treatment center, biopsy method, tumor type, receptor status in breast, histopathological evaluation of lymph node biopsy, date and type of lymph node marking, type of NACT, use of HER2-receptor antagonists, date of surgery, successful identification and re-marking of the MLN at US before surgery, and surgical identification of the MLN. Successful detection of the MLN was defined as finding the MLN based on the marking procedure. The MLN detection also was considered successful if found as an SN or by intraoperative US. The marking procedure was recorded as unsuccessful if the MLN was found by axillary sampling, ALND, at a second surgical procedure following re-marking (third marking procedure), or if the MLN was never found.

In addition, we registered whether the MLN was an SN, the number of SNs removed, nondetection of the SN, histopathological evaluation of the MLN and SNs, and whether the patient received completion ALND, including the number and status of LNs removed in ALND. If needed, the local data collector from each data-providing department could submit comments describing irregularities in the marking procedure or identification of the MLN.

Patients where diagnostic biopsy did not specify the type of carcinoma and who had pCR in the breast and axilla at surgery were classified under “Carcinoma NOS.” Ethnicity data are not routinely collected in Danish medical journals and thus were unavailable.

### Outcomes and Statistical Analyses

The primary outcome was the success of MLN excision stratified by the marking method. Secondary outcomes include the proportion of patients where the MLN also is an SN, nondetection of SN, success rate of TAD, and the proportion of patients achieving ax-pCR. We used descriptive statistics and Pearson’s χ^2^ test with Yate’s continuity correction for comparisons or Fisher’s exact test in case of low expected cell counts. Reported effect estimates and confidence intervals were derived from the χ^2^ or Fisher’s exact test. All tests were two-tailed, and the α-level was set at 0.05. R statistical software calculated all statistics (R Core Team, 2021, Vienna, Austria).^24^

The Danish Data Protection Agency (j.no. P-2019-811), the Danish Patient Safety Authority, and the Center for Regional Development of the Capital Region (j.no. 31-1521-208) approved the project.

## Results

We identified 565 patients from the DBCG database and local lists. Of these, 17 patients met exclusion criteria for the following reasons: 1) five patients had previous ipsilateral cancer; 2) five patients had less than four series of NACT or NACT with palliative intent; 3) three patients had primary MagSeeds^®^ placed as a marker and no re-marking performed; 4) one patient had had ipsilateral SLNB for malignant melanoma in the past; 5) one patient had lymph node marking performed after NACT; 6) one patient had NACT and surgery performed abroad; and 7) one patient declined excision of MLN expressly. Another five patients were excluded from the TAD analysis but were included in the descriptive statistics and analyses of ax-pCR, because they had FNAC of the MLN before surgery, with malignant cells, leading to ALND. Thus, 543 patients were eligible for analysis.

The median patient age was 51 years. Breast tumor histology was invasive ductal carcinoma in 90.3%. Regarding NACT, 97.1% received anthracycline and taxane-based regimens. All patients had a coil placed as the primary lymph node marker. The median time from coil marking to surgery was 170 days. For patient characteristics, see Table 1.

**Table 1:** TAD with various marking methods in 548 Danish breast cancer patients treated 2016-2021

	N (median)	% (range)
Total no. patients	548	100
Age (yr)	(51)	(21–82)
Lymph node biopsy method		
CNB	126	23.0
FNAC	422	77.0
Neoadjuvant chemotherapy regimen		
Anthracycline/taxane combination	532	97.1
Other	16	2.9
Breast Surgery Department		
Rigshospitalet	121	22.1
Herlev Hospital	51	9.3
Zealand University Hospital	113	20.6
Odense University Hospital	36	6.5
Hospital of South West Jutland	47	8.6
Viborg Regional Hospital	36	6.6
Aarhus University Hospital	38	6.9
Aalborg University Hospital	21	3.8
Lillebaelt Hospital	85	15.5
Breast tumor histology		
Invasive ductal carcinoma	495	90.3
Invasive lobular carcinoma	11	2.0
Carcinoma NOS^a^ and other	42	7.7
Receptor subtype		
ER^−^/Her2^−^	108	19.7
ER^−^/Her2^+^	86	15.7
ER^+^/Her2^−^	216	39.4
ER^+^/Her2^+^	138	25.2
Remarking procedure in preparation for surgery		
Hook-wire	263	48.0
Iodine seed	103	18.8
Ink marking on axillary skin	62	11.3
Magnetic marker	3	0.5
No remarking; nonidentification of marked LN on US	87	15.9
No remarking; expect to find coil in SN	19	3.5
No remarking; technically or logistically difficulties	6	1.1
FNAC before surgery with malignant cells leading to ALND	5	0.9
Marking period, days (543 patients)	(170)	(89–299)
Overall ax-pCR	250	45.4
ax-pCR ER^−^/Her2^−^	57	52.8
ax-pCR ER^−^/Her2^+^	71	82.6
ax-pCR ER^+^/Her2^−^	29	13.4
ax-pCR ER^+^/Her2^+^	93	67.4

*TAD* targeted axillary dissection; *CNB* core needle biopsy; *FNAC* fine needle cytology aspiration; *ER* estrogen receptor; *Her2/neu* human epidermal growth factor receptor 2; *LN* lymph node; *US* ultrasound; *SN* sentinel node; *ALND* axillary lymph node dissection; *ax-pCR* axillary pathological complete response; *NOS*^a^ not otherwise specified

### Re-marking before Surgery

Ultrasound-guided re-marking of the coiled LN before surgery was successful in 431 of 543 (79.4%) patients. At re-marking before surgery, 263 patients (48.4%) had a hook-wire placed, 103 patients (19.0%) had an iodine seed, 62 patients (11.4%) had an ultrasound-guided ink marking on the axillary skin, and three patients (0.6%) had a magnetic marker (Magseed^®^) placed (Table 1).

The remaining 112 patients (20.6%) had no re-marking before surgery. In most patients, the reason for nonidentification of the coil-marked LN at the preoperative US was an inability to visualize the coil embedded in the LN (87 patients). The risk of nonidentification of the coil-bearing LN on US was significantly higher when patients had ax-pCR, where the OR was 2.05 (95% confidence interval (CI) 1.26–3.37, *p* = 0.0024) compared with patients without ax-pCR.

### Surgical Excision of MLN

**Fig. 1:**
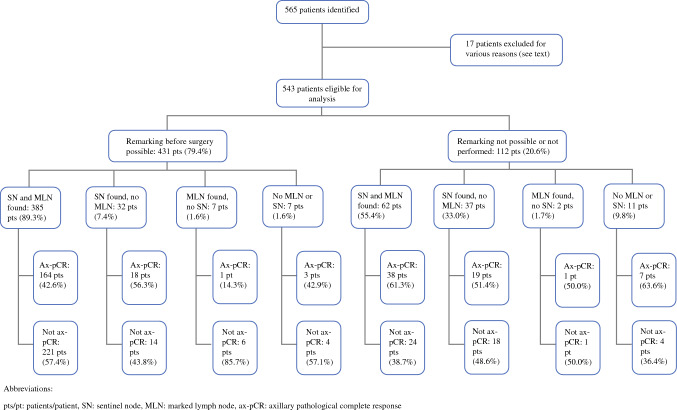
Patient flow and outcomes among Danish breast cancer patients receiving targeted axillary dissection after neoadjuvant chemotherapy

In the 431 patients with a successful re-marking before surgery, surgical excision of the MLN was successful in 392 patients (91.0%) (see Fig. 1 for re-marking success and results of the TAD procedure). Because of coil-marked LNs found by SLNB or intraoperative ultrasound in 25 patients despite unsuccessful marking, the overall IR of the coil-marked LN was 456 of 543 (84.0%). In 39 patients (9.0%) with successful re-marking before surgery, the secondary marking procedure or attempt did not enable surgical excision of the MLN (Table 2). Only iodine seed and magnetic marker as secondary marking procedure reached a surgical excision success of the MLN above 95%.

**Table 2:** Successful excision of MLN after successful secondary marking procedure in 431 Danish breast cancer patients

	Successful excision, n (%)	OR (95% CI)	*p*
Overall	392 (91.0)		
Iodine seed	99 (96.1)	1.00	0.01
Hook-wire	239 (90.1)	2.48 (0.84–7.35)	
Ink marking on axillary skin	51 (82.3)	5.34 (1.61–17.60)	
Magnetic marker	3 (100)	–	

*MLN* marked lymph node

When testing the secondary marking procedures against each other, we found a significantly increased risk of unsuccessful excision when using ink marking on the axillary skin compared with iodine seeds, with an odds ratio (OR) of 5.33 (95% CI 1.62-17.60). Likewise, the risk of unsuccessful marking when using hook-wire was more than doubled compared to iodine seeds (OR 2.48, 95% CI 0.84-7.35), but this difference was not statistically significant.

### Surgical Excision of SN

Excision of at least one SN was possible in 516 of 543 pts (95.0%), resulting in nondetection of the SN in 27 of 543 patients (5.0%). The median number of SNs removed was 2. Excision of three or more SNs was possible in 215 of 543 patients (39.6%). In 402 of 516 patients (77.9%), the MLN displayed signs of tracer and was an SN.

### Results of the TAD Staging Procedure

When combining SLNB with at least one SN excised and excision of MLN, the TAD procedure succeeded in 447 of 543 patients (82.3%) (Table 3). Considering the component procedure’s ability to identify residual tumor burden in the lymph nodes, fifteen patients had MLN metastases despite the SN being without metastases, corresponding to 10.6% of patients where the MLN was not an SN (15/141 patients). For patients where the MLN was without metastases, nine patients had metastases to the SN, corresponding to 1.9% of the 484 patients with known MLN pathology and at least one SN excised.

**Table 3:** Results of the TAD (SLNB + excision of MLN) staging procedure in 543 Danish breast cancer patients

	SLNB success	MLN excision success	TAD success
Identification rate, IR	95.0% (516/543)	84.0% (456/543)	82.3% (447/543)

*TAD* targeted axillary dissection; *SLNB* sentinel lymph node biopsy; *MLN* marked lymph node

Overall, 250 of 548 patients (45.4%) had ax-pCR, with the highest ax-pCR rate in patients with ER−/Her2+ tumors (82.6%) and the lowest ax-pCR rate in patients with ER+/Her2– tumors (13.4%).

## Discussion

In this study, we demonstrate that the chance of successful excision of a coil-marked LN in TAD is fivefold higher when the secondary marker used is an iodine seed compared with marking the axillary skin with ink to guide the incision. Based on these results, ink marking on the axillary skin is not recommended as a secondary marking method when measuring the ability to excise the coil-bearing lymph node based on the secondary marking. The chance of successful excision of the coil-marked LN was 2.5 times higher for iodine seeds than hook-wire, but this was not statistically significant.

With two-step TAD, this study shows that re-marking of the coil-bearing LN is possible in approximately 80% of the patients. Nevertheless, despite successful marking, one in ten patients still have surgical nondetection of the twice-marked MLN. Others also have described the inability to localize and excise the MLN due to nondetection of the coil-bearing MLN on the preoperative US;^25^ nonidentification on the preoperative US amounts to 16.7–67.6%.^18,26,27^ Reasons for surgical nondetection of successfully twice-marked lymph nodes are unclear; one study reported that in 13% of the patients, the markers were found in different LNs.^28^

Reliably identifying the coil-bearing LN and re-marking it for surgery is the cornerstone of two-step TAD, as high procedural feasibility safeguards patients from ALNDs due to nondetection of the MLN. Indeed, we found an approximately twofold increase in odds that the coil-bearing lymph node cannot be found on the preoperative US when the patient has ax-pCR. This underlines the importance of choosing a marking method that is reliably detectable after NACT, because these patients are exactly the patients expected to benefit from TAD.

To our knowledge, this is the first study intending to compare different methods for two-step TAD. Other series have explored the identification rate of the MLN. For two-step TAD with coil and iodine seeds, studies including 25-35 patients show a 91-97% IR of the MLN.^12,21,27^ With coil and hook-wire combination, the reported IR of the MLN is 70.8-98.4% in four series with 23-64 patients.^20,26,29,30^ One of these studies, however, excluded patients with nonidentification on the preoperative US, and the reported IR of 95.7% may be overestimated.^29^ In a different study reporting on TAD with coil and ink marking on the axillary skin, the IR was 84%.^31^ One study, including 37 patients, reported an IR of 81.1% for intraoperative US-guided MLN excision.^32^

Challenging re-marking after NACT in two-step TAD is avoided in the one-step TAD methods. In one-step TAD, the patient has the LN marker placed at the time of diagnosis, and the marker is already identifiable in a surgical setting. Large-scale studies on one-step TAD comprise the RISAS trial demonstrating an IR of the MLN of 94.1% and the TATTOO trial with the surgical IR of the MLN amounting to 91.3%.^33,34^ In our research group, we conducted a one-step, retrospective TAD study with a method like the RISAS trial and found an IR of 99.3%.^35^

None of the two-step methods investigated in our study reached an IR of 99%. This indicates that regardless of marking method, one-step marking seems superior to two-step marking considering surgical reliability, because it negates the need for preoperative US identification and returns an excellent IR. If choosing two-step methods, these results discourage ink marking on the axillary skin solely based on the IR of the MLN. However, patient preference and logistics surrounding surgery might play a role when choosing secondary marking methods. Where ink marking on the skin spares the patients a second, invasive procedure but must be performed immediately before surgery, placing radioactive seeds and hook-wires requires invasive US procedures but differs in flexibility surrounding surgery logistics.

We found that 45% of patients achieved ax-pCR. This result is only slightly higher than the RISAS study (ax-pCR rate 35.4%^33^), a large, Dutch meta-analysis (ax-pCR rate 37%^36^), and the ALLIANCE trial^37^ (41.1% ax-pCR). Rates of ax-pCR vary widely with tumor-receptor profiles.^38^ Thus, selecting patients for NACT may explain differences in reported ax-pCR rates.

In three of four patients, the MLN displayed signs of tracer. A higher count of suspicious nodes on the diagnostic US may increase the likelihood that the coil is placed in a non-SN, because accessibility also plays a role when deciding in which lymph node to place the coil. Caudle et al.'s finding that four or more abnormal lymph nodes on US were associated with the MLN not being an SN support this concept.^11^ Patients with MLN not being an SN warrant increased attention as the risk of a false-negative result in these patients is 16.7-26.9%.^39,40^ Indeed, in our study, 10.6% carried metastases in the non-SN-MLN despite the SN being without metastases. Conversely, less than 2% had metastases to an SN despite an MLN without metastases.

This study has some weaknesses. First, the retrospective nature hinders the collection of information not routinely collected. Retrospective studies also carry some information bias, especially because some breast surgery departments submitted fewer patients for their size than expected. We cannot assess an FNR or NPV, because ALND is discouraged in the case of ax-pCR in Danish guidelines. However, this is an extensive study with 548 included study subjects, enabling us to compare surgical success across different two-step methods—a feat to our knowledge not yet undertaken—thereby adding to the body of evidence on the TAD procedure.

TAD as a research field still contains a pressing knowledge gap for oncological safety studies. We await both the MINIMAX study (clinicaltrials.gov ID NCT04486495) and the AXSANA (clinicaltrials.gov ID NCT04373655) study run by EUBREAST to contribute urgently needed results.^8,41^

## Conclusions

Two-step marking TAD in Danish patients has an 80% success in the identification and excision of the MLN based on the marking procedure across all marking methods currently in use in Denmark. Using ultrasound-guided ink marking on the axillary skin is significantly inferior in surgical ability to excise the coil-marked LN and considering different options should be encouraged. Overall, 45% of the patients achieve ax-pCR, and nonidentification on the preoperative US is more likely in the case of ax-pCR, stressing the need for choosing reliable TAD methods.
